# Sensitivity of commercial pumpkin yield to potential decline among different groups of pollinating bees

**DOI:** 10.1098/rsos.170102

**Published:** 2017-05-31

**Authors:** Sonja C. Pfister, Philipp W. Eckerter, Jens Schirmel, James E. Cresswell, Martin H. Entling

**Affiliations:** 1 Institute for Environmental Sciences, University of Koblenz-Landau, Fortstraße 7, 76829 Landau, Germany; 2 Biosciences, University of Exeter, Hatherly Laboratories, Prince of Wales Road, Exeter EX4 4PS, UK

**Keywords:** *Bombus*, *Apis*, *Cucurbita*, ecosystem services, Halictidae, pollination effectiveness

## Abstract

The yield of animal-pollinated crops is threatened by bee declines, but its precise sensitivity is poorly known. We therefore determined the yield dependence of Hokkaido pumpkin in Germany on insect pollination by quantifying: (i) the relationship between pollen receipt and fruit set and (ii) the cumulative pollen deposition of each pollinator group. We found that approximately 2500 pollen grains per flower were needed to maximize fruit set. At the measured rates of flower visitation, we estimated that bumblebees (21 visits/flower lifetime, 864 grains/visit) or honeybees (123 visits, 260 grains) could individually achieve maximum crop yield, whereas halictid bees are ineffective (11 visits, 16 grains). The pollinator fauna was capable of delivering 20 times the necessary amount of pollen. We therefore estimate that pumpkin yield was not pollination-limited in our study region and that it is currently fairly resilient to single declines of honeybees or wild bumblebees.

## 1. Introduction

Pollination is a valuable ecosystem service, especially for crops requiring animal pollination such as pumpkin [1,2]. Worldwide, 75% of our leading food crops benefit from animal pollination, mainly by bees [1]. Pollination services from wild insects are important, even in the presence of honeybees *Apis mellifera*, because they ensure and enhance pollination through spatial and temporal complementarity, behavioural interactions and higher effectiveness [3–5]. For example, wild bees can be more effective pollinators than honeybees and can increase the fruit set of a wide variety of important cash crops such as almond, spring rape, strawberry, watermelon, cucumber and squash [6]. While numbers of honeybees and wild bees have declined in some areas during the past decades, the demand for insect-pollinated crops has grown [2,7]. Potentially, this may lead to pollination deficits and increases in yield variability [8,9]. In temperate regions, mainly honeybees are used for managed crop pollination outdoors. Reliance on just honeybees increases the risk of uneconomic yields, because it uses only a single species. Furthermore, honeybees are likely to be more susceptible than indigenous wild bees to stressors such as diseases, because the human breeding reduced their genetic diversity [10]. Consequently, a diverse community of wild pollinators can be important for insuring crop yield [11], and it is therefore important to establish whether unmanaged pollinators are alone capable of sustaining pollination services.

We therefore investigated the contributions of different pollinators to fruit set in commercial fields of the pumpkin *Cucurbita maxima* Duchesne ex Poir cv Hokkaido. Insect pollination is essential in pumpkin because all cultivated *Cucurbita* species have unisexual flowers requiring pollen transfer from male to female flowers for fruit set [12]. Although cucurbits have a long flowering period (on average 72–80 days), the single flowers of pumpkin remain open from between 6 h and 1 day. Rapid and effective pollinator visits are therefore vital to maximize yields. To attract pollinators, the *Cucurbita* flowers offer relatively rich rewards of pollen and nectar [13,14]. In Europe, the specialized pumpkin bees (*Peponapis, Xenoglossa*) do not exist [15,16], thus pumpkin flowers could be pollinated by honeybees, bumblebees and halictid bees [13,14,17]. However, the knowledge about the performance of these pollinators has been largely restricted to honeybees [13,18,19] and wild bees in other parts of the world [20–22], and there are no previous studies on Hokkaido pumpkin.

Two main components are required for a quantitative understanding of the relationship between crop yield and the composition of the flower-visiting fauna: (i) the cumulative pollen deposition of each pollinator taxon during the flower's lifespan (further: ‘cumulative pollen deposition’) and (ii) the relationship that links pollen receipt to seed/fruit set [23–25]. Floral visitors vary in cumulative pollen deposition because of variation in both visitation rates and the amount of pollen transferred during a visit [23,26]. By knowing this relationship, the impact of pollinator declines can be predicted and the resilience of food security can be explored. Nevertheless, to our knowledge, this relationship is only known for two economically important crops: cranberry [27] and canola [28]. Both cranberry and canola have bisexual flowers, each with fairly small numbers of ovules (less than 40). In this study, by contrast, we investigated a crop with unisexual flowers and large numbers of ovules (400–700) [13,15]. The separation of male and female flowers confers a high degree of pollinator-dependence on the crop, because mechanisms of autonomous (within-flower) pollination such as seen in canola [28] are impossible, and the large number of ovules initially suggests a need for delivering numerous pollen grains to stigmas.

The aim of this study was to analyse the cumulative pollen deposition of honeybees (*A. mellifera*) and two kinds of wild bee groups, namely bumblebees (mainly *Bombus terrestris agg.*, which include *B. terrestris*, *Bombus lucorum* and rarely *Bombus cryptarum*) and halictid bees (several species, mainly of the genus *Lasioglossum*), as pollinators of pumpkin. Cumulative pollen deposition was characterized by combining pollen deposition per single flower visit with flower visitation rates [25]. We used controlled hand-pollinations to determine the relationship between a stigma's receipt of pollen and the likelihood that the flower set a harvestable pumpkin and the fruit's mass. Based on these data, we modelled the contribution of each pollinator group to crop yield and investigated the potential impact of reductions in bee abundance. The objectives were as follows: (i) to determine the pollination requirements of Hokkaido pumpkins; (ii) to determine the most effective pollinators of Hokkaido pumpkin; (iii) to determine whether there is a pollination deficit in the current pollination system; and (iv) to investigate the sensitivity of crop yield to declines of the three bee groups.

## 2. Material and methods

### 2.1. Study region

We conducted our studies in 2012, 2014 and 2015 in 26 commercial *C. maxima* cv. Hokkaido fields (3 ± 2.6 ha) in the Upper Rhine Valley between Kandel and Ludwigshafen, Germany (49°4 N, 8°6 E; 49°27 N, 8°28 E). The area has a temperate climate with annual mean temperatures around 11°C and 700 mm of annual precipitation on average.

### 2.2. Single visit pollen deposition

According to Ne'eman *et al*. [24], pollinator effectiveness is the contribution of the pollinators to pollen deposition independently of resources spent or available. We measured per visit pollination effectiveness via single visit pollen deposition (SVD). SVD was investigated in three different fields between 8 July and 23 August 2015. SVD on the stigma was measured for honeybees *A. mellifera* (*n* = 43), bumblebees *B. terrestris* agg. (*n* = 42) and halictid bees (Halictini, size: 5–10 mm, more than 50% *Lasioglossum malachurum*, *n* = 33). These three groups of bees were chosen, because they are the main flower visitors of pumpkin in our region. Honeybees most likely originated from apiaries in the region, but no hives were found within 70 m of the pumpkin fields. Flowers were bagged prior to anthesis and again after the single visit with a synthetic mesh bag (mesh size ≈1 mm^2^) to exclude further pollinator visits. For each replicate, one single bee was allowed or engineered to visit one virgin bagged female flower. Flowers were left on the plants and the observer waited for a bee to visit the flower (allowed) or caught a bee and released it at the flower's corolla (engineered, halictids). As few halictid bee visits occurred naturally in the studied fields, we performed additional replicates in August and engineered their visits by catching them from a male flower and transferring them to a virgin female flower. The duration and the time at which the visit occurred was noted. We tried to evenly space the observed visits over the approximately 4 h interval of flower receptivity in our experiments between 06.45 and 10.45. After pollinating, the halictid bees were caught when they left the flower and later identified in the laboratory, where their length and intertegular span were measured. The stigmas of the experimental flowers were cut and frozen for later quantification of pollen numbers. In order to quantify pollen removal from anthers, we measured the number of pollen grains present in open and bagged flowers over daytime, which we used to estimate the overall efficiency of the pollen transfer system. In August 2012 and 2015, the anthers of eight bagged flowers (2012) and of 44 open flowers (2012: 13; 2015: 31) were taken between 07.30 and 11.00 for later quantification of pollen numbers.

### 2.3. Flower visitation rate and handling time

We studied flower visitors and their foraging behaviour in 18 fields in our study region in 2014. Each field was investigated three times, once in each one time period during the flowering period (2–6, 15–17, 23–25 of July 2014), and once at 07.00, 08.30 and 10.00, respectively. On each occasion, we recorded four 15 min long videos each surveying a different female pumpkin flower. The camera, a digital HD video camera recorder (handycam Sony® HDR-CX115E), was positioned approximately 50 cm above a female flower in order to monitor the mouth of the flower's corolla. Video recording is a suitable method to sample visitation rates in pumpkin [20,29], because the frequency of visits is high and relatively evenly distributed across female flowers. From the videos, we extracted for each bee group the visitation rates and their flower handling time (*H* = the entire duration the bee spent on and in the flower, from landing until leaving). Three bee groups were distinguished: (i) honeybees *A. mellifera*, (ii) bumblebees = *B. terrestris* agg. and *Bombus lapidarius* were identified from the videos, and (iii) halictid bees. Halictid bees could be distinguished only into two size-defined groups (length ≈ 6 mm and length ≈ 8 mm), each containing several halictid species. Additionally, we recorded for each visit the time of day, the elapsed time spent at the nectaries and whether or not the insect contacted the flower's stigma.

### 2.4. Relation of fruit set and yield to pollen deposition

To determine the relation of fruit set and yield (seed set and fruit mass) to pollen deposition, we conducted controlled hand-pollination experiments for three reasons. First, the pollen loads delivered by bees can vary greatly. Second, the SVD of a single bee may not be sufficient for fruit set. Third, potential fruit and seed set can be highly reduced through abortion, especially in plants with floral overproduction like pumpkins [24,30,31]. Hand-pollination experiments were conducted in one field per year (2014, 2015). Female flowers were bagged the day before anthesis with a synthetic mesh bag (mesh size ≈1 mm^2^). At anthesis, they were hand-pollinated and re-bagged. Hand-pollination was done between 7.00 and 11.00 to ensure pollen viability and stigma receptivity. Stigmas are normally receptive until 13.30 [18] and although pollen viability decreases during anthesis, we predict it to be 75% at 13.00 based on a previous study [13]. We always pollinated the first female flower of a plant to avoid enhanced abortion rates through first-fruit dominance, thereby maximizing the chance of measuring seed set. Each pumpkin plant produces several consecutive female flowers during the flowering period. The first female flower is the flower that blooms as the first in the flowering period. In 2015, we removed non-experimental fruits from the treated plants at intervals of 1, 3 and 6 or 7 days after the pollination of our focal flower in order to minimize abortions among the hand-pollinated fruit [31].

For transferring different amounts of pollen to the stigma, we initially (2014) created five levels of pollen deposition by dissecting single anthers into parts of different sizes (electronic supplementary material, table S1). Given the high variability of pollen numbers in deposits obtained with this method, we changed the method in 2015, when we used a metal wire (tip diameter 1 mm) or a nail head (diameter 2.4 mm) in several repetitions and combinations to transfer seven levels of pollen to stigmas (electronic supplementary material, table S1). Experimental pollinations at each level were replicated 20 times in 2014 and around 30 times in 2015. At the beginning of September, the pumpkins were harvested from the experimentally pollinated flowers and the fruit mass plus the number of fully developed seeds were measured. Our main measure of crop yield is the proportion of fruit set, but we also investigated fruit mass because Hokkaido pumpkins are sold for human consumption. After consultation with local farmers, fruits with a minimum weight of 800 g were defined as marketable.

### 2.5. Quantification of pollen numbers

In order to quantify pollen deposition, we extracted the pollen from each stigma by acetolysis following Jones [32]. After acetolysis, glycerol 50% was added to the extracted pollen to a total volume of 0.5 ml. All pollen from the stigmas from the single visit experiments was counted under ×65 magnification.

In order to determine the amount of pollen in anthers, the pollen was washed off the anthers with 70% ethanol. After the pollen grains had sedimented by centrifugation, the supernatant was removed with a micropipette and glycerol 50% was added to the pollen pellet to make up 5 ml (in 2012) and 1 ml (in 2015) (based on Vidal *et al*. [33]). To evenly re-suspend the pollen, the vials were shaken by a vortex mixer prior to counting the pollen in 10 (bagged anthers) or five (open anthers) subsamples of 20 µl in 2012. In 2015, the pollen was counted in three to nine subsamples of 50 µl (depending on the standard deviation of the counted pollen). The total pollen load of each male flower was estimated volumetrically from the mean of the subsamples.

### 2.6. Data analyses

**Figure 1.**
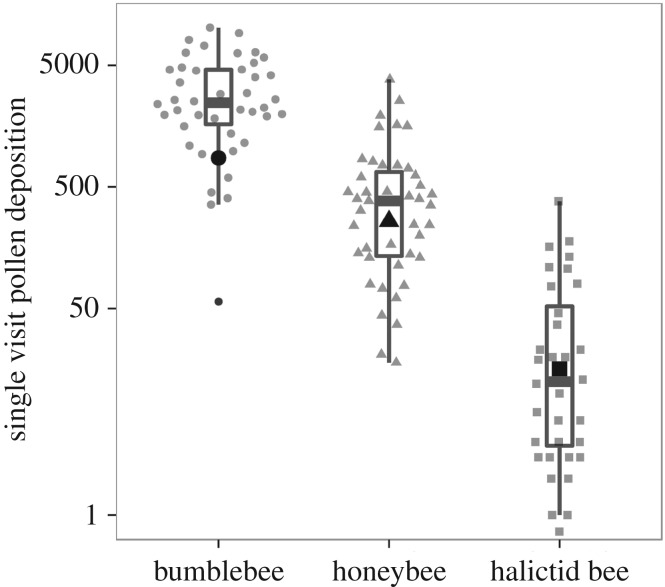
The number of pollen grains deposited in single visits to flowers by the pollinator groups in our study, or SVD. For each bee group, the box plots present the median, quartiles and range in the conventional style and the accompanying scatter depicts the individual observations. For realism in our model, we calculated weighted averages, denoted $\bar{d\;}\;\;$, to account for variation in handling times, in size, in stigmatic contact and in pollen transfer over the flowering interval. For each pollinator, the values of $\bar{d\;}\;\;$ are shown as a large circle (bumblebees), a triangle (honeybees) and a square (halictid bees). The small black circle below the data for bumblebees marks an outlier that we excluded from our analyses.

All statistical analyses were conducted in R v. 3.2.2 [34]. In order to determine whether per visit pollination effectiveness varied among the pollinator groups, we used multiple pairwise comparisons using the method of Herberich *et al*. [35] to account for the heterogeneous variances and unbalanced group sizes (R packages ‘multcomp’, ‘sandwich’). For each bee group, we tested the following potential explanatory variables for SVD: length (only for halictid bees), handling time, time of day of visit and their interactions, and we dropped non-significant terms (*p* > 0.1) from final models. The SVD data were log-transformed to reduce the heterogeneity of variance. To account for non-normality, we checked the *p*-values with permutation tests (R package ‘pgirmess’). Best models were selected using Akaike's information coefficient (R package ‘MASS’). In the SVD dataset, one outlier (probably a technical anomaly, bumblebee; figure 1) was removed prior to data analysis.

In order to determine whether cumulative pollen deposition varied among pollinator groups due to differential rates of flower visitation, we tested whether the response variables of the video data, i.e. handling time (log-transformed) and visitation rate, varied among bee groups using multiple pairwise comparisons. For the comparison of the handling times, we used the method of Herberich *et al*. [35] to account for the unbalanced group sizes (see above). In the comparison of the visitation rates, we included ‘field’ as random factor.

To test whether the amount of pollen available at the anthers of flowers declined during anthesis, we used a linear mixed-effect model on a combined dataset with ‘year’ (levels of 2012, 2015) as a random factor (R packages: ‘nlme’, ‘piecewiseSEM’).

In order to relate crop performance to pollen deposition (*D*), we used the data from the hand-pollination experiments to describe the dependence of fruit set of harvestable (i.e. not aborted) pumpkins on pollen deposition (log-transformed), which was tested by fitting a nonlinear three-parameter (*a*, *b* and *c*) logistic model with the following form:

2.1 $$F=\frac{a}{100\times (1+\exp[(-\log10(D)+b)/c])},$$

where *F* denotes the proportion of harvestable fruit. As fruit set is a binary variable, we used a binomial distribution to model the statistical error in the proportion of harvestable pumpkins. In order to further investigate the basis for variation in fruit mass among the fruits that were set, we used the data obtained from hand-pollinations to evaluate whether fruit mass depended on the estimated deposition of pollen (log-transformed) and the numbers of fully developed seeds.

### 2.7. Modelling the contribution of bee groups to crop yield

We assume that the probability that a flower produces a marketable pumpkin depends on the amount of pollen accumulated on its stigma. In order to model the cumulative pollen deposition of a certain bee group *i*, let *v_i_* denote the visitation rate of bee group *i* (visits flower^−1^ h^−1^) during the flower lifetime of *R* h. Let each single visit by bee group *i* deposit *d_i_* pollen grains. The contribution to pollen accumulation of bee group *i*, *D_i_*, is therefore given by:

2.2 $$D_{i}=v_{i}Rd_{i}.$$

We relate crop performance (i.e. the proportion of harvestable pumpkins), *F*, to the expected total pollen accumulation *D* using the sigmoidal relationship described above (equation (2.1)). To model the effect of a specified pollinator decline, we introduce a proportional change in the visitation rate of bee group *i* in equations (2.1) and (2.2). In the analyses below, we assume that the flower is receptive to pollination for *R* = 4 h.

In order to estimate the expected rate of pollen deposition due to each pollinator group, we had to account for the following four circumstances (see Results): (i) the magnitude of SVD (of bumblebee and honeybee) decreased as the flowers aged; (ii) for bumblebees, the magnitude of SVD increased with the time spent handling the flower, denoted *H*, and the handling times were much shorter in realistic situations (*H* = 12 ± 23 s) than in the single visit experiments (*H* = 151 ± 64 s; *t* = −29.5, *p* < 0.001); (iii) for halictid bees, the SVD varied with length of the individual bee, denoted *l* and (iv) only a proportion of floral visits result in contact with the flower's stigma, denoted *s*. For modelling purposes, we therefore calculated the expected pollen deposition for a single pollinator visit in equation (2.2), *d_i_*_,_ as a weighted average for each pollinator group using only significant terms from statistical analyses (see Results) as follows.

Bumblebees:

2.3 $${\bar{d\;}\;\;}_{B}=s_{B}\underset{T}{\sum }[V_{T}(10^{\left(\alpha -\beta T+\gamma H\right)})],$$

where ${\bar{d}}_{B}$ denotes the expected number of pollen grains delivered to a flower's stigma by a bumblebee visit, *s*_B_ denotes the proportion of floral visits in which bumblebees contacted the flower's stigma, *H* indicates the time spent handling the flower, *T* indicates that we separately treated the approximate 4 h lifetime of the flowers (between 07.00 and 11.00) as five sequential segments (each of 48 min, so that the values of *T* are the decimalized times: 0.31, 0.34, 0.38, 0.41 and 0.44). Values for the unsampled second and fourth intervals (i.e. *T* = 0.34 and *T* = 0.41, respectively) were calculated as mean of the two adjacent segments, either the first and third or the third and fifth, respectively. *V_T_* denotes the proportion of bumblebee visits that occurred in time interval *T* in the video data (from *T*_1_ to *T*_5_: 0.08, 0.15, 0.21, 0.26, 0.31). Fitted constants from statistical analyses (see Results) are denoted by *α*, *β* and *γ*.

Honeybees:

2.4 $${\bar{d\;}\;\;}_{A}=s_{A}\underset{T}{\sum }[{\bar{V}}_{T}(10^{\left(\alpha -\beta T\right)})],$$

where ${\bar{d\;}\;\;}_{A}$ denotes the expected number of pollen grains delivered to a flower's stigma by a honeybee (*Apis*) visit, with symbols annotated as for equation (2.3).

Halictid bees:

2.5 $${\bar{d\;}\;\;}_{H}=\underset{l}{\sum }[s_{l}V_{l}d_{l}],$$

where the proportion of visits due to each of two length classes, *l* = 1 and *l* = 2, are denoted by *V_l_*, the size-specific probability of stigma contact is denoted *s_l_* and the per visit pollen deposition of each size class is denoted by *d_l_*. Specifically, the halictid bees were separated into two length classes as follows: *l = *1, comprising individuals ≈ 6 mm long (i.e. *Lasioglossum morio, L. pauxillum* and *L. politum*); and *l = *2, comprising individuals ≈ 8 mm long (mainly *L. malachurum*). The small halictid bees (*l*_1_) deposited only eight pollen grains per visit (s.d. ± 8.2, *n* = 10), whereas larger halictid bees (*l*_2_) deposited around 46 pollen grains (s.d. ± 56.5, *n* = 15).

We estimated the mass of fruit produced per hectare of crop using the following model:

2.6 Y=FNMP,

where *Y* denotes the yield of Hokkaido pumpkins in tonnes per hectare, *F* is the probability that a flower sets a harvestable pumpkin (related to pollen deposition using equation (2.1)), *N* denotes the number of female flowers per plant, *M* denotes the mass of a single Hokkaido pumpkin (t) and *P* denotes the number of plants (individuals ha^−1^). For our calculations, we assumed: *N* = 6 female flowers per plant based on field observations on 30 plants; *M* = 0.001 tonne per fruit because 1 kg is the optimal weight for the market; and *P* = 10 000 plants ha^−1^ based on responses to farmer questionnaires (*n* = 35 fields, data not shown). Furthermore, we assume that the same number of female flowers bloom each day.

## 3. Results

### 3.1. Cumulative pollen deposition

#### 3.1.1. Single visit pollen deposition

Bumblebees deposited almost six times more pollen grains per single visit (mean ± s.d., SVD =3369 ± 2473, *n* = 41) than honeybees (SVD = 582 ± 752, *n* = 43; *t* = 9.11, *p* < 0.001) and 75 times more than halictid bees (SVD = 45 ± 76, *n* = 33; *t* = 16.8, *p* < 0.001). Honeybees deposited 13 times more pollen than halictid bees (*t* = 8.96, *p* < 0.001; figure 1). The pollen deposition that resulted from a bumblebee visit (*grains *= 10^(3.906 + 0.0027*H* − 2.565*T*)^; *R*^2 ^= 0.24) increased with handling time (*t*_38_ = 3.34, *p* = 0.0019) and tended to decrease as the day progressed (*t*_38_ = −1.85, *p* = 0.072; electronic supplementary material, figure S1a). The pollen deposition of a honeybee visit decreased significantly over the course of the morning (*grains* = 10^(4.44–5.54*T*)^; *t*_41_ = −3.4, *p* = 0.0015, *R*^2^ = 0.20) (electronic supplementary material, figure S1*a*). The pollen deposition of a halictid bee visit tended to increase with body size (grains = 10^(0.168 + 0.170*l*)^ − 1*; t*_27_ = 2.0, *p* = 0.052, *R*^2 ^= 0.10).

Bagged male *C. maxima* flowers contained on average 37 000 pollen grains (s.d. = 6900, *n* = 8). In open male flowers, the number of pollen grains remaining on the anthers decreased to approximately 600 by 11.00 (*grains* = 10^(6.9–9.0*T*)^; *t*_41_ = −11.0, *p* < 0.001; $R_{\mathrm{marginal}}^{2}=0.53$) (electronic supplementary material, figure S1*b*), which implies that 98% of pollen was removed before flowers senesced.

#### 3.1.2. Flower visitation rate and handling time

In 54 h of video footage, we observed a total of 2100 individual flower-visitors, of which 79% were honeybees *A. mellifera*, 14% bumblebees (mainly *B. terrestris agg.,* some *B. lapidarius*) and 7% halictid bees. The rate of flower visits by honeybees (123 visits/flower lifetime) was significantly higher than by bumblebees (21 visits/flower lifetime; *t* = 18.5, *p* < 0.001) and halictid bees (11 visits/flower lifetime; *t* = 20.2, *p* < 0.001). The handling time at individual flower visits was very variable, but differed significantly among bee groups. Bumblebee visits (mean ± s.d., *H* = 12 ± 23 s) were more than 10 times shorter than visits of honeybees (*H* = 144 ± 252 s; *t* = 22.4, *p* < 0.001) and of halictid bees (*H* = 191 ± 225 s; *t* = 15.8, *p* < 0.001). Virtually, all visits by bumblebees (*s* = 99%) and honeybees (95%) resulted in contacts with the stigma. We could not visually verify the contact with the stigma by halictid bees while they were descending and ascending the flower, but we assume that they did make contact if they reached the nectaries. Overall, 82% of all halictid bees reached the nectaries during their visit.

### 3.2. Relation of fruit set and yield to pollen deposition

**Figure 2.**
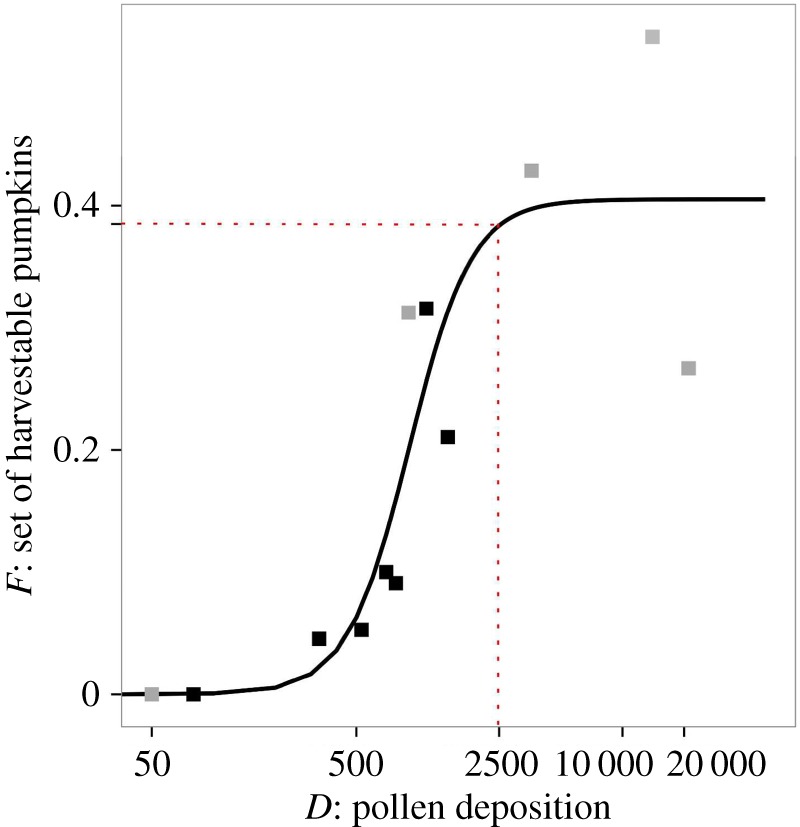
The probability of fruit set of harvestable Hokkaido pumpkins (*y*-axis: *F*) increased with the number of pollen grains deposited on a flower's stigma (*x*-axis: *D*) based on the hand-pollination results from 2014 (grey squares) and 2015 (black squares). The fitted relationship is based on equation (2.1) (see text). According to this relationship, 95% of the maximum level of fruit set (*a* = 41%) occurs when approximately 2500 pollen grains have been deposited on a flower's stigma (dashed lines).

The likelihood that a flower produced a harvestable pumpkin increased sigmoidally with pollen deposition (equation (2.1): *a* = 40.52, *t*_9_ = 7.53, *p* < 0.001; *b* = 2.96, *t*_9_ = 36.5, *p* < 0.001; *c* = 0.15, *t*_9_ = 1.77, *p* = 0.111; *R*^2^ = 0.79; figure 2) and the relationship saturated at a fruit set of 41%. When around 2500 pollen grains were deposited, 95% of this maximum fruit set was obtained (figure 2). Marketable fruits, which weigh more than 800 g, contained at least 140 fully developed seeds and were pollinated with more than 500 pollen grains. Fruit mass (g) increased with the number of fully developed seeds (*mass* = 424.8 + 1.503 *seeds*; *t*_36_ = 3.7, *p* < 0.001, *R*^2 ^= 0.25) and tended to increase with increasing pollen deposition (*mass* = 311.8 + 122.3 × log_10_(*pollen grains*); *t*_36_ = 1.8, *p* = 0.077, *R*^2 ^= 0.06).

### 3.3. The contribution of bee groups to crop yield

**Figure 3.**
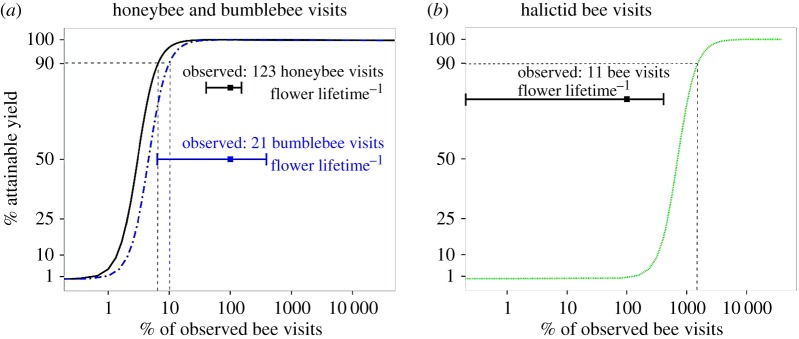
The impact of pollinator decline or increase on yield in Hokkaido pumpkin. (*a*) The estimated attainable yield (*y*-axis: percentage of harvestable pumpkin fruits relative to extant levels) in relation to the rate of flower visits per flower lifetime (*x*-axis: rate of visits as a percentage of extant intensity) by either honeybees (solid line) or bumblebees (dashed line-dotted). (*b*) As for (*a*) except the relationship is for halictid bees. In both panels, a horizontal bar shows the range of the observed visitation rate and the associated filled square indicates the mean. The number of visits corresponding to 100% of the observed visits in the *x*-axis differs per bee group and is displayed above the horizontal bars. Dashed lines indicate the percentage of the extant visit rate that is required to produce 90% of the currently attainable yield (i.e. 7% for honeybees, 11% for bumblebees and 1100% for halictid bees).

During a single flower visit, an average bumblebee, honeybee or halictid bee deposits an expected number of pollen grains of ${\bar{d\;}\;\;}_{B}=864$, ${\bar{d\;}\;\;}_{A}=260$ or ${\bar{d\;}\;\;}_{H}=16$, respectively (electronic supplementary material, table S2). Using these as values for *d_i_* and the current rates of flower visitation in equation (2.2) yields the following estimates of the pollinating capabilities (grains deposited per flower lifetime) of the bee groups: honeybees, 31 980 grains; bumblebees, 18 144; and halictid bees, 183 (electronic supplementary material, table S2). Using these values in conjunction with the pollen-yield relationship (i.e. equations (2.1) and (2.6)) indicates that 90% of attainable crop yield requires a cumulative pollen deposition equivalent to 11% of the extant bumblebee intensity (=2 bumblebee visits/flower lifetime) or 7% of the extant honeybee intensity (=8 honeybee visits) or 1100% of the extant halictid bee density (=123 halictid bee visits; figure 3). Our model predicts that crop yield will be more sensitive to declines of bumblebee than honeybee visits, because a reduction in one bumblebee visit results in the delivery of 600 fewer pollen grains than the reduction in a single honeybee visit (electronic supplementary material, table S2). The model also predicts that the loss of any single pollinator group will not reduce crop yield in our study system. Based on our assumptions, our model predicts a maximum pumpkin yield of 24.3 t ha^−1^ (equation (2.6)), which closely matches the maximum value that is widely reported by farmers in our study area (25 t ha^−1^). The system's potential transfer efficiency is approximately 17% (100 × 50 307/(8 × 37 000)), when the following values are used: the model's predicted pollen deposition by the extant pollinator fauna (50 307 grains; electronic supplementary material, table S2); the number of pollen grains eventually removed from a male flower's anthers (37 000); and the eightfold preponderance of male flowers in our study area (S.C.P. 2012, 2014, 2015, personal observation).

## 4. Discussion

We found bumblebees to be the most effective pollinators per flower visit of Hokkaido pumpkins in Germany, and crop yield is therefore most sensitive to declines in bumblebee visits. However, based on our model, honeybees deposited overall more pollen per flower owing to their greater rates of flower visitation, and pumpkin yield was not pollination-limited in our study region at the extant abundance of bees.

### 4.1. Pollination requirements of Hokkaido pumpkins

We established that the minimum pollination requirements of each flower in *C. maxima* Hokkaido pumpkin were 500 pollen grains for a marketable fruit and that the likelihood of fruit set reached 95% of the maximum with around 2500 pollen grains present on the stigma. Thus, for maximum seed set, approximately four pollen grains per ovule are necessary, which corresponds fairly closely with Cruden's Rule [16,36]. Furthermore, *C. maxima* Hokkaido pumpkins have higher thresholds for fruit set than *Cucurbita pepo* (minimum for fruit set 70 pollen grains, maximum rate of fruit set approx. 1300 pollen grains; [19–21]) and *Cucurbita foetidissima* (minimum 50 pollen grains, maximum greater than 900 pollen grains; [16,37]) and in contrast with other *Cucurbita* species, fruit set does not reach 100% even under optimal pollination [38]. Unlike smaller cucurbit fruits like squash, Hokkaido pumpkins and other larger pumpkins may have higher pollination requirements, but fail to achieve a fruit from every flower even when these are met, because the individual plants lack sufficient resources to invariably produce a marketable fruit [39]. The high pollination requirements of Hokkaido pumpkin relative to other cucurbits underline the need for abundant and stable pollinator populations in their production areas.

### 4.2. Cumulative pollen deposition

Similar to other crops pollinated by honeybees and bumblebees [6,11], bumblebees were the most effective pollinators of pumpkin per flower visit, probably for three reasons. First, bumblebees transfer the most pollen owing to their large body size [4,21] and densely hairy coat [40]. Second, the faster handling of bumblebees relative to the other pollinator groups increased their relative effectiveness: in the same time, a honeybee visits one pumpkin flower a bumblebee could visit 12 flowers (similarly [20,41]). In general, smaller bee species have longer handling times, probably because of their lower nectar extraction rate owing to the shorter proboscis length and the lower body mass [26]. Third, bumblebees, like the specialized squash bees (tribe *Eucerini*) that pollinate pumpkin in the Americas, and in contrast to honeybees and halictid bees reliably touch the reproductive parts of the flower with their ventral side as they handle the flowers, thereby avoiding the attachment of pollen to the head and eyes, which apparently otherwise slows a bee's progress [20,42]. We also observed that pollinator visits were most effective early in the morning when more pollen was available at the anthers for transfer by bees ([19]; electronic supplementary material, figure S1). Furthermore, all investigated pollinators only collected nectar, but did not harvest pollen in male flowers. Despite the eightfold preponderance of male flowers in our study area, our analysis indicates that the extant pollinator fauna was capable of generating a transfer efficiency of approximately 17%. Previously, estimates in systems with friable pollen have reported transfer efficiencies in the region of 1% [43]. In our case, however, we are dealing with an extraordinarily high level of flower visitation with rates in the range of one visit by a honeybee or bumblebee every 2 min (electronic supplementary material, table S2). This high flower visitation results in our high modelled potential for pollen deposition. It is likely that real pollen deposition is somewhat more limited, however, because the stigma's surface becomes eventually saturated with pollen. Thus, when the cumulative pollen deposition is high enough to cause stigma clogging, pollen transfer per bee visit may become increasingly poor later in the flower's life, which is later in the morning in our case. Taken together, these findings are consistent with our observation that fruit production in our Hokkaido pumpkin system was very far from pollen-limited.

### 4.3. Sensitivity of pumpkin yields to bee declines

Our model reveals the relative importance of the components of the bee fauna as follows: 90% of the attainable yield is reached with two bumblebee visits per female pumpkin flower or eight honeybee visits. Thus, in our region, bumblebees provide a substantial ecological service by playing a key role in pumpkin pollination, despite their lower densities relative to honeybees. As individuals, bumblebees are also more effective pollinators than honeybees owing to their faster handling of flowers. However, at the measured visitation rates, honeybees deposit more pollen per flower than bumblebees. Halictid bees do not appear to be capable of pollinating *C. maxima* effectively. It is likely that the pollen deposition of halictid bees is limited by their size [4,22] and hairiness [40] and that their cumulative pollen deposition was actually low. However, our findings must be treated with caution, because our handling of the bees during engineered visits may have affected measurements of pollen deposition. For example, although the handling times of halictid bees were similar in natural and engineered visits (data not shown), the handling during ‘engineered’ visits might have dislodged pollen from the bees. In any case, even a twofold error in our estimate of single visit deposition would not affect our conclusion that halictids barely contributed to the pollination of pumpkin in our study area and that only a many-fold increase (approximately 10-fold) in their abundance would satisfy the pollination requirements of pumpkin. Thus, our findings suggest that social bees are essential for pumpkin pollination in the study region.

Our model predicts realistic values of yield in pumpkin, which suggests that it can be plausibly used to investigate the consequences of changes to the pollinator fauna. On this basis, we predict that current crop yields are sustainable even in the event that any single pollinator group is lost. Consequently, pumpkin could continue to be a profitable crop at our study area despite a catastrophic loss of only honeybees or bumblebees, for example. Thus, our cropping system demonstrates a high level of ‘attack tolerance’ [44] and that wild bumblebees provide ecological insurance [11]. However, even if the causes for the decline of one group of bee would not directly affect other groups of bees as well, removing one type of bee could alter the visitation rate and therefore pollen deposition by other bees.

Bumblebee and honeybee are clearly the key to pollination success of pumpkins in Germany, underlining that crop pollination is often delivered by a few common species in intensified agricultural landscapes [45]. However, we recognize that functional group diversity of bees might nevertheless be important elsewhere. For example, seed set of *Cucurbita moschata* in Indonesia only increased with functional group diversity (25 species, eight functional groups) and not with the number of bee visits [4]. Furthermore, flower-visitor richness increases yields in pollinator-dependent crops worldwide [46].

Our model's predictions should be considered alongside some caveats. For example, our model did not include the pulsed bloom that characterizes most crops. Thus, it is possible that more bees may be needed to successfully pollinate all flowers at the peak flowering time than our model predicts. Second, we have not considered how the probability of fruit set may vary with plant age. In general, the probability of setting a harvestable pumpkin in the first female flower of a pumpkin plant is higher, because pumpkins produce at least twice as many female flowers than fruits, which means that the successful pollination of the first pollinated female flowers reduces the plant's pollination requirements [30]. Based on observations of 30 non-manipulated plants, the probability of setting a harvestable pumpkin in the first female flower of a plant (80%) could be twice as high as the probability of fruit set in all flowers (41%). Third, the threshold of fruit set in the manipulated yield experiments (41%) was much lower than could be expected, given the use of the first female flowers and removal of other flowers. Most probably, because the plants in this experiment received less water than the flowers of the non-manipulated plants, but other differences between fields and Hokkaido cultivars might also have contributed to the differences between fruit sets. On the other hand, the similarity of the calculated threshold of fruit set derived from our yield experiment and the overall probability of fruit set in non-manipulated plants in another field enabled us to combine the probability equation of fruit set derived from our manipulated plants with field data from non-manipulated plants to estimate yield per hectare. Nevertheless, our yield estimates are not precise owing to the possible variation of all input variables. While these additional complexities could be incorporated in future models if desired, we do not anticipate that they would qualitatively change the outcome of our analysis.

### 4.4. Management implications

Importantly, pumpkin received more than enough visits of honeybee and bumblebee in our region. Thus, the system is currently resilient to the decline of either honeybees or bumblebees, but not to the decline of both. However, it should be taken into account that in our region, cucurbit crops are grown in moderate field sizes (3 ha), that they comprise only a small proportion of all cropping area (on average 9 ha pumpkin in 1 km radius) and that few other pollen and nectar resources were available to bees during the bloom of the pumpkin fields (S.C.P. 2012, 2014, 2015, personal observation). Thus, pollinators were probably attracted to the floral rewards in the pumpkin fields from a relatively large area. It is possible that more bees will be needed in other landscapes where the cultivation area of cucurbit crops or competing simultaneously flowering crops is larger. For crops with high pollinator dependence such as pumpkin, yield variability is quite high (approx. 13.2%) [9]. Thus, high pollination levels must be ensured to increase yield stability [9]. Therefore, we recommend a management strategy for pumpkin that supports and sustains high densities of bees. Especially, bumblebees should be supported, because their abundance depends on undisturbed natural land offering nesting sites and year-round floral resources, which are not always available near crops in agricultural settings [5,47]. In intensively farmed areas, the pollinator fauna could be supported by both a high frequency of interspersed semi-natural habitats and areas of organic farming, which can benefit bees by providing flower resources and suitable nesting habitats [7,48].

Finally, we encourage the adaptation of our quantitative approach to other pollinator-dependent crops such as almonds, citrus and apple in order to determine their resilience to potential pollinator decline in different regions. If widely adopted, these techniques could provide mechanistically supported inferences about food security in pollinator-dependent crops worldwide. Overall, studies like ours could become increasingly important for directing stewardship efforts involving habitat management, landscape modification and the protection of bee habitats within the agricultural landscape.

## Supplementary Material

Hand pollination methods

## Supplementary Material

Performance indicators of the Hokkaido pumpkin pollination system and its component pollinators

## Supplementary Material

Influence of time of day on single visit deposition and pollen supply in male flowers

## Data accessibility

All data and code to reproduce this analysis are available from the Dryad Digital Repository: (http://dx.doi.org/10.5061/dryad.fh078) [49].
