# Supplementary material for: Sensitivity of commercial pumpkin yield to potential decline among different groups of pollinating bees
Source: R Soc Open Sci. 2017 May 31;4(5):170102. doi: 10.1098/rsos.170102 (PMC5451820; doi:10.1098/rsos.170102)
Supplement: Hand pollination methods [file rsos170102supp1.docx]

# Electronic supplementary material 1

**Hand pollination methods**

**Table S1** Methods used in 2014 and 2015 to transfer fixed pollen loads to the stigma in the yield experiment. Pollen loads were transferred by different methods: a metal wire (= clip, diameter 1 mm), a nail head (diameter 2.4 mm) and pieces of anthers. Pollen loads were measured on extra flowers, not used for the yield experiment, but treated with the same hand-pollination methods. Given are the replicates of pollen measures and separately replicates of the different levels of pollen numbers in the yield experiment and the mean and standard deviation (SD) of pollen deposited on the stigma. For levels, where we have not measured the transferred pollen, we estimated it from the known data (indicated by “~”). Repetitions of the clip method exactly resulted in multiplicated amounts of transferred pollen, therefore estimates for repetitions and combinations with the clip method were calculated by simple addition and multiplication. Repetition of the nail head method did result in much lower transferred pollen amounts than would be estimated by the multiplication of the amount tranferred by using the nail head once, therefore estimates for the repeated use of the nail head (three and four times) were calculated by multiplication of the measured pollen amount transferred by using the nail head twice. Hand-pollinations for yield measurement were done on 6 days in 2014 (12.- 18.07.2014) and 7 days in 2015 (28.6.- 2.7.2015 and again 21.7. – 1.8.2015, because almost all fruits of the first pollination round in 2015 were aborted owing to heat and water stress independently of the treatment). Per day 10 – 40 plants were hand-pollinated, thus 2 to 6 replicates of each level of pollen numbers per day. If all fruits of all treatments, that were pollinated on the same date, aborted, the data was removed from the statistical analysis (data of 1 day in 2014 and of 2 days in 2015).

| **year** | **method** | **Replicates** | **Transferred pollen** | | **Replicates** |
| --- | --- | --- | --- | --- | --- |
|  |  | Pollen measure | mean | SD | Yield experiment |
| 2014 | counting | 4 | 50 | 0 | 13 |
| 2015 | 1x clip | 5 | 82 | 22 (27%) | 19 |
| 2015 | 3x clip | 4 | 246 | 21 (9%) | 0 |
| 2015 | 4x clip | 0 | ~330 |  | 22 |
| 2015 | 1x nail head | 16 | 528 | 75 (14%) | 19 |
| 2015 | 2x nail head | 5 | 702 | 167 (24%) | 20 |
| 2015 | 1x nail head+ 3x clip | 0 | ~780 |  | 22 |
| 2015 | 3x nail head | 0 | ~1100 |  | 19 |
| 2015 | 4x nail head | 0 | ~1400 |  | 19 |
| 2014 | 1 mm of ¼ anther | 4 | 888 | 549 (62%) | 16 |
| 2014 | 4 mm of ½ anther | 4 | 3575 | 585 (16%) | 14 |
| 2014 | 1 anther | 4 | 14038 | 4818 (34%) | 13 |
| 2014 | 5 anthers | 4 | 21088 | 12391 (59%) | 15 |
