# Supplementary material for: Sensitivity of commercial pumpkin yield to potential decline among different groups of pollinating bees
Source: R Soc Open Sci. 2017 May 31;4(5):170102. doi: 10.1098/rsos.170102 (PMC5451820; doi:10.1098/rsos.170102)
Supplement: Performance indicators of the Hokkaido pumpkin pollination system and its component pollinators [file rsos170102supp2.docx]

# Electronic supplementary material 2

|  |  |
| --- | --- |

**Performance indicators of the Hokkaido pumpkin pollination system and its component pollinators**

1. **Table S2** Performance indicators of the Hokkaido pumpkin pollination system and its component pollinators: bumblebees, honeybees, and two sizes of halictid bees. The figures show the taxon-specific contributions to the total estimated number of pollen grains (50307) that are deposited on an average flower’s stigma by 155 flower visits with annotation indicating model solutions or parameter values.

| Name | Parameter | Bumblebee | Honeybee | Small halictid | Large halictid |
| --- | --- | --- | --- | --- | --- |
| Total deposition | *D_i_* | 18144 | 31980 | 48 | 135 |
| Per visit deposition | *d_i_* | 864 | 260 | 6 | 45 |
| Visits per flower lifetime | *v_i_R* | 21 | 123 | 8 | 3 |
| Stigmatic contact | *s* | 0.99 | 0.95 | 0.76 | 0.98 |
| Handling time | *H* | 12 | 144 | 153 | 298 |
| No. of observations | *n* | 282 | 1664 | 114 | 40 |
