# Supplementary material for: Sensitivity of commercial pumpkin yield to potential decline among different groups of pollinating bees
Source: R Soc Open Sci. 2017 May 31;4(5):170102. doi: 10.1098/rsos.170102 (PMC5451820; doi:10.1098/rsos.170102)
Supplement: Influence of time of day on single visit deposition and pollen supply in male flowers [file rsos170102supp3.docx]

# Electronic supplementary material 3

|  |  |
| --- | --- |

**Influence of time of day on single visit deposition and pollen supply in male flowers**


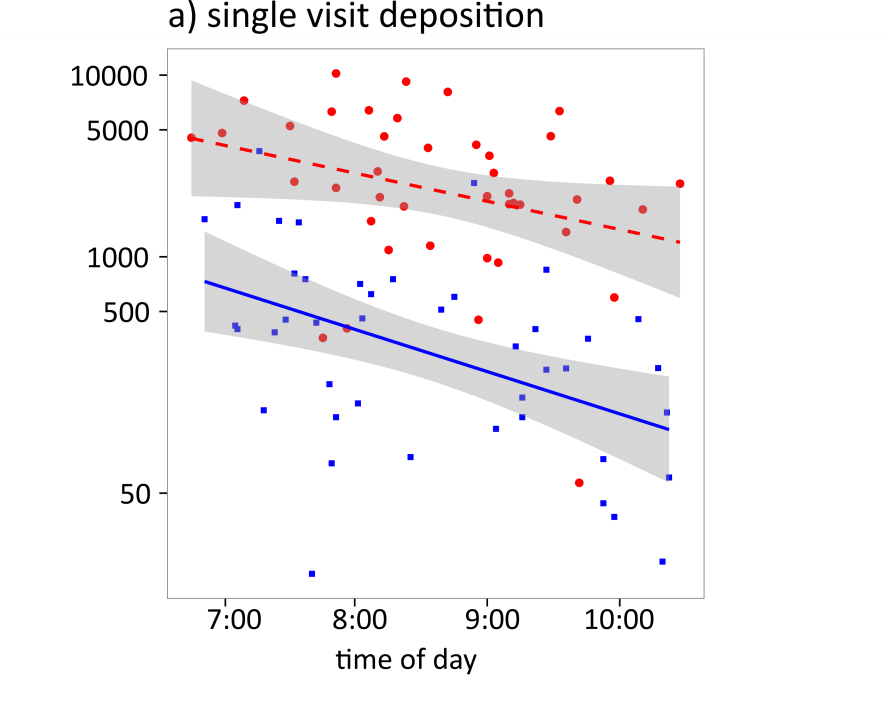


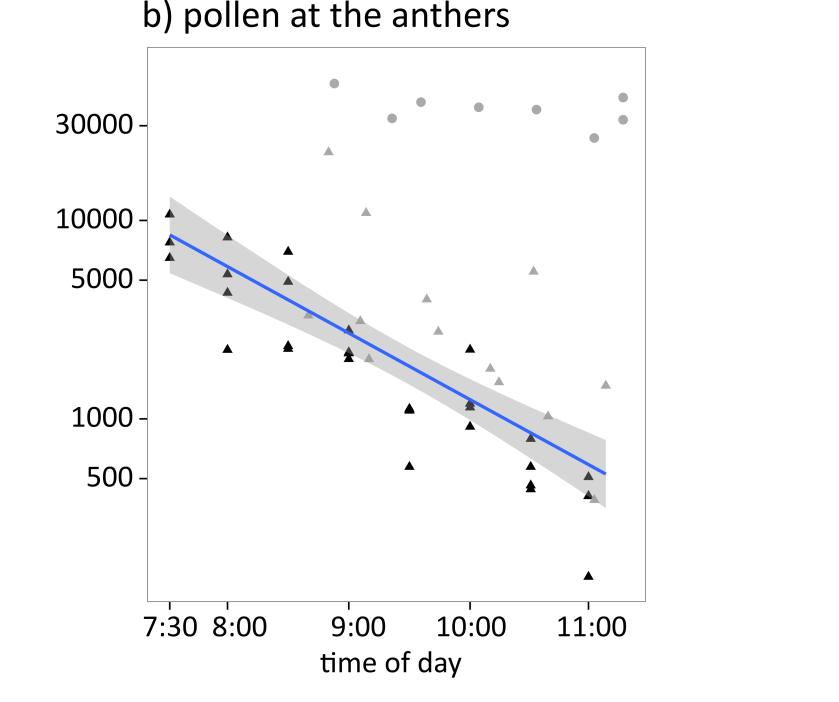


1. **Fig. S1** The availability of pollen in the anthers of male flowers and pollen deposition on the stigmas of female flowers decreased over the daily flowering interval.

a) Single visit pollen deposition on the stigma by honeybees (squares, solid line) and bumblebees (circles, dashed line) decreased in the course of the morning.

b) Number of pollen grains at the anthers in open male flowers decreased in the course of the morning (triangles). The initial amounts of pollen in closed male flowers (circles) did not vary over time and was c. 37000 pollen grains. Datasets collected in two separate years are distinguished by colour (2012: grey, 2015: black).

First visitors remove a high amount of pollen (see also a) pollen deposition), thus male flowers can loose a high amount of pollen very early in the morning. In 2015 male flowers were visited by around 3 bumblebees and 12 honeybees per hour.
